# Artificial Intelligence-Based Approaches to Reflectance Confocal Microscopy Image Analysis in Dermatology

**DOI:** 10.3390/jcm11020429

**Published:** 2022-01-14

**Authors:** Ana Maria Malciu, Mihai Lupu, Vlad Mihai Voiculescu

**Affiliations:** 1 Department of Dermatology, Elias University Emergency Hospital, 011461 Bucharest, Romania; ana.malciu@gmail.com; 2 Department of Dermatology, Carol Davila University of Medicine and Pharmacy, 050474 Bucharest, Romania

**Keywords:** dermatology, dermoscopy, in vivo, confocal microscopy, deep learning, artificial intelligence, skin cancer, artifact

## Abstract

Reflectance confocal microscopy (RCM) is a non-invasive imaging method designed to identify various skin diseases. Confocal based diagnosis may be subjective due to the learning curve of the method, the scarcity of training programs available for RCM, and the lack of clearly defined diagnostic criteria for all skin conditions. Given that in vivo RCM is becoming more widely used in dermatology, numerous deep learning technologies have been developed in recent years to provide a more objective approach to RCM image analysis. Machine learning-based algorithms are used in RCM image quality assessment to reduce the number of artifacts the operator has to view, shorten evaluation times, and decrease the number of patient visits to the clinic. However, the current visual method for identifying the dermal-epidermal junction (DEJ) in RCM images is subjective, and there is a lot of variation. The delineation of DEJ on RCM images could be automated through artificial intelligence, saving time and assisting novice RCM users in studying the key DEJ morphological structure. The purpose of this paper is to supply a current summary of machine learning and artificial intelligence’s impact on the quality control of RCM images, key morphological structures identification, and detection of different skin lesion types on static RCM images.

## 1. Introduction

In vivo reflectance confocal microscopy (RCM) has demonstrated, time and again, to be an important imaging tool for dermatologists. This technology offers a non-invasive view of the skin structure similar to histopathology, revealing tissue architecture and individual cells [1,2] down to about 200–250 μm from the skin surface [3]. Even though, at the moment, dermoscopy is the most widespread method for skin cancer screening, RCM has demonstrated significantly higher diagnostic accuracy in several skin tumors [1,4,5,6,7]. RCM has a sensitivity and specificity of more than 70% for identifying skin cancers [8,9,10,11,12,13,14,15], as well as for diagnosing and monitoring the evolution of inflammatory or other skin diseases [8,16]. RCM has also been used for lateral excision margins mapping before the surgical treatment of skin tumors. This technique increases the odds for complete tumor excision, lowering the recurrence rate [17]. Furthermore, when dermoscopy is used in conjunction with RCM, the number of unnecessary biopsies can be reduced [7].

RCM is a suitable bridge between dermoscopy and histopathology, allowing clinicians to do bedside, non-invasive, virtual skin biopsies in real-time [18]. Depending on the source of contrast, imaging can be done with fluorescence or reflectance method. Ex vivo confocal microscopy, in its recently improved version, which includes digital staining, has successfully been used to guide Mohs surgery [19].

RCM images may be difficult to interpret because, unlike dermoscopy and histopathology, there are fewer training programs available for RCM. This method is not included in the regular dermatology training curriculum. Coupled with the morphological nature of RCM, this may lead to subjective variability in confocal-based diagnoses.

Research has shown that a well-coached physician can identify basal cell carcinomas with 92–100% sensitivity and 85–97% specificity [9,12,14,17], and melanomas with 88–92% sensitivity and 70–84% specificity [10], using in vivo RCM imaging. However, those very high figures, can be attained only by doctors who have used this device for five to ten years and have trained to interpret these images efficiently. Several deep learning algorithms have been developed over the last five years to assess RCM images quantitatively [9,20,21].

Machine learning-based algorithms, it is hypothesized, could offer a more quantitative, objective approach [22]. Computer image examination tools for skin lesion monitoring were previously used with dermoscopy images and dermatopathology slides to improve diagnostic accuracy [4,23,24]. Without requiring the clinician’s input, automated diagnostic tools report a score representing observed malignant potential [4]. Several algorithms, including image segmentation, statistical techniques, and artificial intelligence, have been used [8].

This paper aims to show a more detailed overview of the machine learning-based algorithms used for RCM image analysis and diagnostic discrimination. The review brings together the different concerns of confocal image analysis addressed through machine learning, the methods used so far, and offers a clear view of the path ahead.

## 2. Materials and Methods

A Google Scholar and PubMed search was performed on 10 July 2021 to identify published reviews, clinical trials, and letters to the Editor related to AI use and RCM, without enforcing a time period. The adopted search strategy was identical for both databases and implemented using the keywords: “confocal microscopy”, “artificial intelligence”, “skin”, and “machine learning”. Thus, the authors gathered the latest data reported in the literature about the influence of machine learning algorithms on the RCM images quality control, key morphological structures identification, and detection of different skin lesion types on static RCM images.

## 3. Results

### 3.1. Quality Assessment of Reflectance Confocal Microscopy Composite Images (Mosaics)

Even though in vivo RCM is becoming more common in modern dermatological practice, there are instances when the diagnosis is not immediate and the images are evaluated after the patient has left the office. In these cases, the patient must return to the clinic if additional imaging is required, to wit, due to poor quality images being recorded.

**Figure 1:**
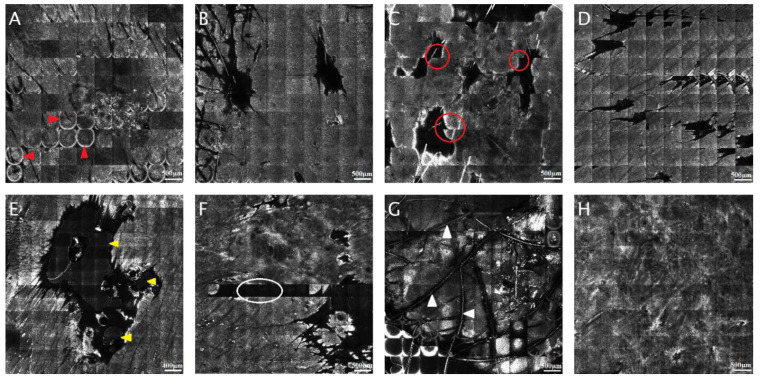
The most common reflectance confocal microscopy (RCM) mosaic artifacts. (**A**) Circular hyper-reflective rings caused by corneal layer reflection (red arrowheads). (**B**) Bright contour of each RCM picture (5 × 5 mm). (**C**) Sliding of single RCM images (5 × 5 mm); notice how there is no proper alignment of the images on different rows (red circles). (**D**) Repeating images in the RCM mosaic (5 × 5 mm). (**E**) Air and oil bubbles (yellow arrowheads). (**F**) Black individual images (white circle) without useful information in the RCM mosaic (5 × 5 mm). (**G**) Hair and bright relics (white arrowheads). (**H**) Correctly captured RCM mosaic. (RCM images courtesy of M.L.).

The most frequent artifacts seen in RCM images are relics related to reflection on the surface. That can appear in RCM mosaics due to the presence of corneal layer reflection in the form of hyper-reactive rings, or it can occur as a lighter contour of every single RCM mosaic compared with the rest of the image. Another type of artifact that shows the shifting and misalignment of sole RCM mosaics can be induced by the person’s movement, respiratory movements, talking, or even the heart beating. In the case of papules or nodules, bright or dark relicts appear in the region where the microscope and the skin are not touching [25]. In addition, in some RCM mosaics, air and oil bubbles can be seen commonly stuck by skin creases, hair follicles, or surface unevenness throughout the investigation. Occasionally, several individual images of RCM mosaics are underexposed or completely clear. Sometimes can appear changes in contrast due to the microscope’s automatic intensity control and several curved lines traversing the image, representing hair fibers. These artifacts are widespread, but, as long as they do not affect the entire mosaic rendering it unreadable, the images can still be helpful. Figure 1 shows the most common artifacts that appear in RCM mosaics.

To prevent this from happening, layers of quality assurance are critical in the clinical setting, at the moment of acquisition to obtain good pictures, as well as at the moment of analysis, to reflect the chances of a good specimen [26]. Automated RCM image quality assessment could also play a major role in ensuring a more streamlined workflow, as the examiner can go through images and cases at a faster pace if mosaics are of consistently good quality. Moreover, less experienced RCM users would also benefit from only looking at high-quality confocal mosaics. This would facilitate recognition of structures and criteria, thus speeding up their learning process.

Bozkurt et al. [27] illustrated the use of the MUNet network to segment RCM images into morphological patterns. This architecture can be comprised of several nested U-Net networks. U-Net is a convolutional neural network (CNN) designed exclusively for segmenting medical pictures. This system relies entirely on a CNN, but its structure has been adapted to perform with a reduced number of pictures and output accurate segmentation [28,29]. In this study, the authors assessed and categorized images of melanocytic skin lesions obtained at the dermal-epidermal junction (DEJ) for six patterns: background, artifacts, meshwork pattern, ringed pattern, clod pattern, and non-specific pattern. The proposed algorithm’s sensitivity and specificity for artifact detection were 79.16% and 97.44%, respectively. Although this outcome appears encouraging, preparing the network entails using a training set as underlying data.

In another research by Kose et al. [26], an identical network was used to design a method for automatically evaluating the quality of RCM mosaics. To recognize and measure unhelpful areas of RCM mosaics, the authors agreed to use the RCM image and the corresponding dermoscopy images. They categorized artifacts, as well as regions outside of lesions, as uninformative areas. The model’s sensitivity was 82% and its specificity was 93%. This research showed that image analysis based on machine learning technology could reliably detect unclear regions in RCM images. It also inferred that implementing machine learning-based picture quality evaluation could be valuable for creating quality requirements, as well for workflow streamlining. This would be achieved firstly during image capture, lowering patient recall due to poor quality images being recorded, and, secondly, during picture analysis, to aid in determining the probability of non-representative selection.

At a later date, a multi-scale MED-Net neural network was presented as an improvement of the MUNet idea by Kose et al. [30]. The MED-Net algorithm was designed to differentiate diagnostically relevant patterns (e.g., meshwork, ring, nested, non-specific) in RCM images at the DEJ of melanocytic lesions from non-lesional and diagnostically uninformative areas. The network was used to divide RCM mosaics into six categories, one of which was artifacts. The system achieved an artifact detection sensitivity of 83% and a specificity of 92%. Although this outcome was somewhat satisfactory, the network still required expert data labeling. Table 1 summarizes the sensitivities and specificities of the various techniques used to identify artifacts in RCM images.

Based on a carefully monitored end-to-end machine learning technique, Wodzinski et al. [31] developed a method for evaluating if a provided RCM image has a reasonable quality for subsequent lesion classification. In this deep learning method, all of the different parts were trained simultaneously, rather than sequentially. This study presented an attention-based network to handle the challenge of high-resolution imagery while requiring a tiny group of network variables and minimal inferential complexity. The approach worked across a wide range of skin diseases and represented an important element in the diagnostic process. It could also help collect information directly by notifying the operator about image quality instantaneously throughout the evaluation. The accuracy on the test set was greater than 87% [31].

Although ensuring the quality of acquired RCM images is paramount for diagnostic precision and workflow optimization, assisted interpretation tools, such as automatic, ideally real-time, identification of specific landmarks and structures, are welcome additions. These tools have been in research for some time, closely following in the footsteps of previous research focused on automated analysis of dermoscopy and histopathology images.

### 3.2. Automated Delineation of the Dermal-Epidermal Junction

**Figure 2:**
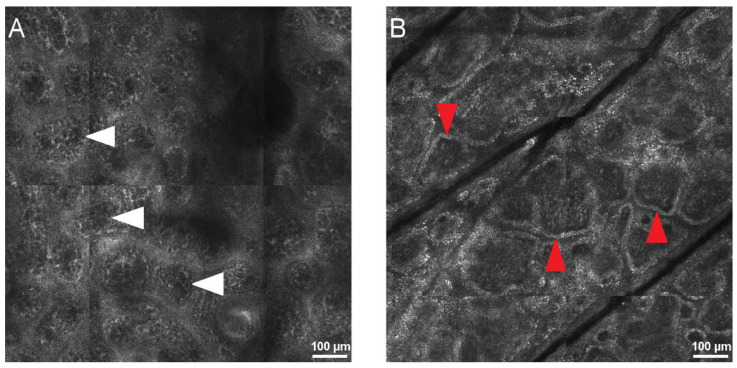
Confocal aspect of the normal dermo-epidermal junction (DEJ) in human skin. (**A**) RCM mosaic (800 × 800 µm) at the DEJ in Fitzpatrick phototype I skin, showing dermal papillae that are not defined by hyper-reflective rings (white arrowheads). (**B**) RCM image (800 × 800 µm) at DEJ in Fitzpatrick phototype III skin, revealing bright rings around dermal papillae (red arrowheads) determining the ringed papillae pattern. RCM images courtesy of M.L.

DEJ identification during RCM image analysis is important for diagnosing both skin lesions, such as melanoma or melanoma in situ, type lentigo maligna, and inflammatory skin, such as lupus erythematosus, lichen planus, bullous diseases, and psoriasis. The visual method for identifying the DEJ in RCM images is subject to interpretation, resulting in a wide range of outcomes [22]. Furthermore, while it is easily detected in darker phototypes, the DEJ can be difficult to identify in fair skin and hypo- or depigmented lesions (Figure 2). Techniques based on artificial intelligence can supply an extra analytical view and perform a preliminary examination of acquired RCM images.

In a paper from 2015, two methods that can autonomously demarcate the DEJ in RCM images of regular skin were tested [22]. To adjust for misalignment caused by patient motion during imaging, this automated approach began by recording the images in every stack sideway. Because these methods depend on changes in regional tile-specific qualities with depth, this step was critical. The technique generated a mean error of 7.9 ± 6.4 μm in a dark complexion, where the contrast was high due to melanin. The algorithm defined the DEJ as a passing area in fair skin, with a median error of 8.3 ± 5.8 μm for the epidermis-to-passing zone limit and 7.6 ± 5.6 μm for the passing zone-to-dermis edge.

Kurugol et al. [32] introduced a hybrid segmentation/classification technique that divides z-stack tiles into uniform segments by adapting the model to dynamic layer patterns, then classify tile segments as epidermis, dermis, and DEJ by structural features. To achieve this, designs from three fields were combined: texture segmentation, pattern classification, and sequence segmentation. The research looked at two various learning circumstances: one for using the same stack for training and testing, and the second for training on one designated stack and testing on a different subject with comparable skin type. The DEJ is distinguishable in both situations with epidermis/dermis misdiagnosis rates below 10% and a mean range from expert-labeled limits of around 8.5 μm.

High melanin backscatter induces the pigmented cells located over the DEJ to have a lighter appearance in hyperpigmented skin. The algorithm operated on small regions to find the peaks of each tile’s smoothed average intensity depth profile to pinpoint these bright areas. The results revealed that this method properly identified the skin phototypes for all 17 examined images. The DEJ recognition algorithm obtained a mean length from the underlying data DEJ of approximately 4.7 μm for dark complexion and nearly 7–14 μm for pale skin [33].

Kose et al. [21] presented a deep learning model that can mimic a clinician’s subjective and graphic process of examining RCM images of the DEJ. The mosaics were divided into regional processing spaces, and the algorithm identified the textural appearance of each space using dense Speeded Up Robust Feature (SURF). These features were used to teach a support vector machine (SVM) classification model to differentiate among meshwork, ring, clod, specific, and background patterns in different skin lesions. The outcomes of 20 RCM images classified by a specialist in RCM reading demonstrated that these patterns could be classified with 55–81% sensitivity and 81–89% specificity.

Hames et al. [34] reported an algorithm for learning a per-pixel fragmentation of a full-depth RCM image with poor surveillance using labels for the whole en-face segments. This algorithm was created and evaluated on a set of data with a total of 16,144 en-face segments. This method correctly identified 85.7% of the testing dataset, and it was capable of recognizing underlying modifications in skin layers depths of aged skin.

### 3.3. Convolutional Neural Networks and Classification and Regression Trees for Skin Lesion Identification on Static RCM Images

A unique class of algorithms, defined as a neural network, and, more particularly, a version identified as a deep neural network, has been used in most recent advances in diagnostics. Small computer programs called neural networks take in pieces of information and analyze them to produce hypotheses. Neural networks are designed for a specific function utilizing examples with default results. The neural networks then make predictions relying on these samples, which are then assessed for accuracy. The convolutional neural network is the most commonly used deep neural network model for image examination [35].

Artificial intelligence algorithms, such as CNN, have been previously used successfully in dermatology for image analysis [35] and have shown particular promise in medical image classification [31,36,37].

**Figure 3:**
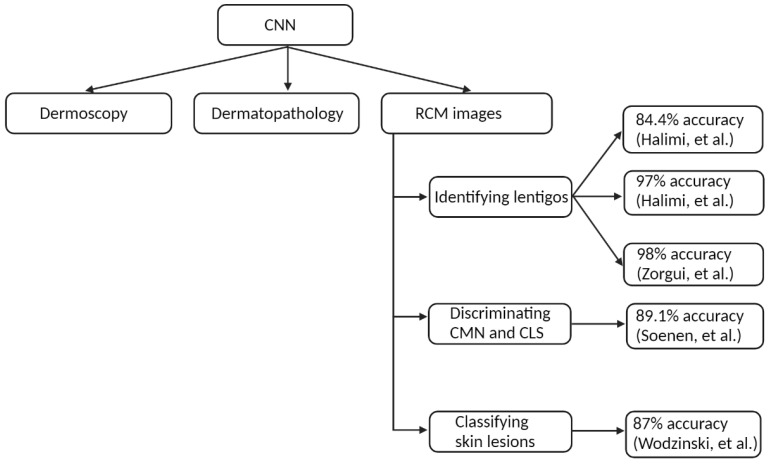
The use of convolutional neural networks in dermatology. CNN: convolutional neural network; RCM: reflectance confocal microscopy; CMN: congenital melanocytic nevus; CLS: café au lait spot [36,48,49,50,51].

In dermatology, CNN has been extensively tested, with varying degrees of success, to analyze dermoscopy images [38,39,40,41,42,43] and digitized slides in dermatopathology [35,44,45,46,47]. As a natural progression, interest grew in the utility of deep learning in RCM image analysis (Figure 3).

For example, one study [48] aimed to identify and characterize lentigos. RCM images are high-resolution images taken at various skin depths. In this study, the researchers used a double wavelet decomposition technique. The wavelet coefficients were then subjected to quantitative analysis with the help of a SVM to sort RCM pictures into lentigo or clear skin, with an accuracy of 84.4%.

A novel Bayesian technique for both restoration and categorization of RCM pictures was reported by Halimi et al. [49]. This method that distinguished clear skin from lentigo had a 97% accuracy rate. In this paper, the authors offered two RCM lentigo detection approaches that have proven to be complex and difficult to implement in the past, needing manual processes, such as feature selection and data preparation.

Zorgui et al. [36] suggest a novel 3D RCM picture recognition approach for lentigo diagnosis. A CNN InceptionV3 architecture was used to create the technique [52]. The InceptionV3 is a complex and highly developed network with solutions that improve precision and speed without stacking countless layers. This network was trained on 374 pictures and then tested on 54 images. RCM images were classified into healthy and lentigo skin with 98% accuracy. The proposed CNN method has enormous potential and yields very promising results.

**Figure 4:**
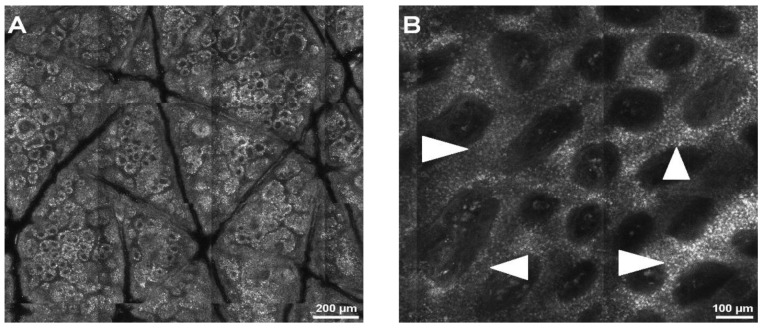
Normal skin and lentigo appearance during RCM examination. (**A**) RCM image (1.5 × 1.5 mm) at the dermal-epidermal junction (DEJ), showing normal skin architecture. (**B**) RCM mosaic (1 × 1 mm) at the DEJ, displaying a lentiginous pattern through thickening of the inter-papillary spaces (white arrowheads), an aspect typical for lentigo. RCM images courtesy of M.L.

Halimi et al. [53] described a low-complexity recognition technique to determine the skin profoundness where a lentigo might be identified. This approach decomposed the image acquired for each skin layer into multiple resolutions. This method was tested on 45 patients with normal skin and with lentigos from clinical research (2250 images in total), yielding a sensitivity of 81.4% and a specificity of 83.3%. The findings revealed that lentigo could be found at depths of 50 to 60 μm, which corresponds to the DEJ’s average location (Figure 4).

Soenen et al. [50] studied the differences between the several types of congenital pigmented macules using RCM images and machine learning techniques. Three experts evaluated the images in a blinded manner. Then, using RCM images at the level of the DEJ, a pre-trained CNN combined with a SVM algorithm was used to identify café au lait spots and congenital melanocytic nevi. Café au lait spots could be distinguished from congenital melanocytic nevi based on unique features on RCM pictures, according to the results. Machine learning can aid pattern recognition and improve the accuracy rate in congenital melanocytic nevus differentiation on RCM images. Furthermore, RCM proves to be a valuable non-invasive diagnostic method in challenging cases and in patients with congenital melanocytic nevi with equivocal dermatoscopic appearances.

**Figure 5:**
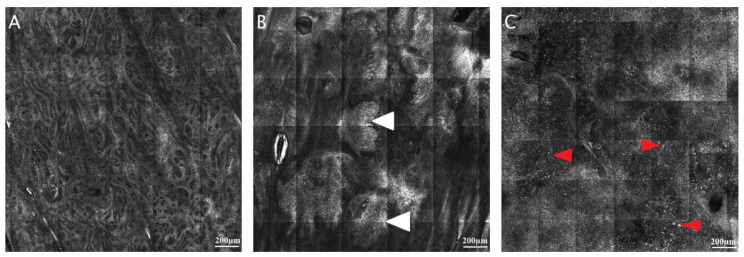
Confocal aspect of different skin tumors in reflectance confocal microscopy (RCM) images. (**A**) RCM mosaic (2 × 2 mm) at the dermal-epidermal junction (DEJ), showing the meshwork pattern of a benign melanocytic nevus. (**B**) RCM mosaic (2 × 2 mm) at DEJ, showing large, hyper-reflective tumor islands (white arrowheads) connected to the epidermis through tumor cords in a pigmented, superficial basal cell carcinoma. (**C**) RCM mosaic (2.5 × 2.5 mm), showing complete disarray of the DEJ and massive infiltration by large, round, atypical cells (red arrowheads) in cutaneous melanoma. RCM images courtesy of M.L.

Wodzinski et al. [51] proposed a method for classifying skin lesions using RCM mosaics based on a CNN. The ResNet architecture was used to build the proposed network. First, the network was trained on the ImageNet dataset. The dataset contained 429 RCM mosaics grouped into three categories: melanoma, trichoblastic/basal cell carcinoma, and melanocytic nevi (Figure 5), with the diagnosis validated through histology evaluation. The detection precision of the test set was 87%, which was significantly greater than that of participating confocal users. These findings suggest that the presented categorization method could be a helpful technique for noninvasively detecting beginning melanoma.

The use of artificial intelligence techniques for evaluating different melanocytic skin lesions on RCM images was investigated by Koller et al. [1]. The ‘Interactive Data Language’ software tool created the image analysis procedure. Features of image analytics were based on transforming the wavelet. Salford Systems’ CART (Classification and Regression Trees) analytic software executed the classification method. CART begins by growing the greatest tree feasible for each tree, then cuts the parts of the tree that do not play an important role in the precision rate up to the base node. The teaching sequence was utilized to create a classification tree initially. The unbiased data was then used to confirm the obtained tree model in a second stage. CART accurately labeled 93.60% of the melanoma and 90.40% of the nevi pictures in the training group. When the whole dataset of pictures was used, the CART tree identified 58.48% of the melanoma and 48.51% of the nevi. The classification achievement of a blinded, objective, and neutral observator extremely skilled in RCM was also studied in all the grouping method test images. The independent clinical dermatologist correctly classified 78.95% of the melanocytic lesions (62.50% of the melanoma and 84.50% of the moles). Future research could benefit from these computerized RCM image recognition techniques [1].

Kaur et al. [54] showed that a CNN trained on a medium-sized image database has poor accuracy. They presented a hybrid deep learning method that trains a deep neural network using traditional text-based feature vectors as input. The dataset contained 1500 images from 15 RCM stacks representing six separate skin layer components. The study showed that this hybrid deep learning approach outperforms CNN in terms of test accuracy, with a score of 82% versus 51% for CNN. They also compared their results to other RCM image recognition algorithms and found this method more accurate.

## 4. Discussion

Proper analysis of biomedical images is an important element in the diagnostic process of many illnesses recognized through certain images, such as histopathology and radiology [55]. Multiple non-invasive methods, such as RCM, optical coherence tomography (OCT), non-linear optical (NLO) microscopy, multiphoton microscopy (MPM), and dermoscopy, are previously used in dermatology to facilitate the diagnosis of different skin conditions [56].

Compared to microscopy techniques, OCT has the advantage of deeper penetration (several hundred microns), but it is still limited in evaluating cellular morphologic features. These techniques use different contrast modes to identify specific molecular compounds in images [57]. Unlike RCM, NLO penetrates more profound and can distinguish between different elements of the dermal connective tissue [58]. In comparison to RCM, MPM techniques can successfully identify morphological, structural, and even chemical information of both skin cells and extracellular matrix without external labeling. The vascular structures can also be easily observed using fluorescence labeling [59]. These novel techniques are used to assess transdermal drug delivery, disease diagnosis, cancer diagnostic, extracellular matrix abnormality, and hair follicle pathology [60]. In recent years, many studies on lesion diagnosis using dermoscopy images and AI showed a better performance for deep learning methods than dermatologist diagnostic accuracy [61]. The use of dermoscopy in conjunction with RCM could improve the diagnostic accuracy of melanocytic lesions, demonstrating the value of complementary techniques [62,63].

Other non-invasive diagnostic methods used in dermatology other than RCM can see the general architecture of the skin and penetrate three times more profound into the skin than RCM. RCM, on the other hand, provides higher resolution images that can identify different individual cells and their characteristics with three times greater detail. These higher resolution images aid machine-learning algorithms in identifying various skin cells and diagnosing skin disease [64,65,66].

RCM is a promising non-invasive imaging technique that allows the assessment of histological skin and lesion details at the epidermal and upper-dermal levels, thus lowering the need for conventional skin biopsies. Lately, many artificial intelligence algorithms have been developed to provide a more objective approach to RCM image reading. This effort is much needed due to several factors contributing to a relatively high degree of subjectivity and inter-observer variability regarding sensitivity, specificity, and diagnostic accuracy associated with RCM. Machine learning techniques provide great developments, alternatives, and assistance to help dermatologists improve their daily practice.

To date, applications of AI in RCM image analysis have mostly been used to assess the quality of RCM mosaics, identify the DEJ, and help identify and discriminate between different cutaneous tumors. The algorithm types and their utility have been summarized in Table 2.

Quality assessment of RCM images is very important for lowering the number of artifacts, shortening evaluation times as the RCM reader does not waste time accessing irrelevant images, as well as reducing the number of patient visits to the clinic due to insufficient or low-quality images. Regarding this, the progress made so far has been promising, with the sole caveat that the networks still require expert data labeling.

It is a well-known fact for confocal users that pinpointing the DEJ on dark skin is simple due to the significant hyper-reflectivity of basal keratinocytes. At the same time, this can prove a difficult task in fair skin due to the lack of the natural source of contrast, melanin. The algorithms proposed in studies directed at the automated DEJ delineation on RCM images could be a valuable training instrument to aid novice RCM readers in learning the key morphological patterns of the dermal-epidermal junction, thus making RCM a more accessible and widely embraced technology in dermatology clinics. Furthermore, this research suggests that automated algorithms could increase objectivity during real-time in vivo RCM examination by quantitatively guiding the DEJ delineation. The advancement of such algorithms could help evaluate unusual morphological features, especially ones located at the DEJ.

All things considered, there are still gaps in artificial intelligence-based RCM image analysis. Clinical studies that demonstrate the value of AI-based RCM image analysis, along with huge sequence differentiating judgment stages, are still lacking.

Several aspects still need to be improved since there is hardly any data on the achievement of AI in identifying atypical melanocytic lesions, such as those on the scalp or mucosal surfaces. The hair follicle, vessels, and air bubbles are all limitations in the segmentation methods, while acral areas have not been thoroughly assessed yet. Additionally, these techniques still do not include amelanotic melanomas.

In addition, the risk of “deskilling” the dermatologist needs to be addressed. Who will be held accountable for the assessment: the doctor or the brand that created the device? What will be done to establish specificity, and what actions are needed to recognize amelanotic tumors?

## 5. Conclusions

Research showed that most of these artificial intelligence technologies already had reached clinician-level precision in the identification of various skin diseases, and, in some studies, machine accuracy even surpassed them. Thus, further research into the automated RCM image analysis process appears promising. Yet, existing algorithms cannot be used for routine skin cancer screening at this time. As a result, developing a systematic imaging procedure may help to improve the outcomes. Automated image analysis systems provide clinicians with objective and speedy support, while not interfering with the diagnostic process. However, more studies are needed to improve the applicability of automated RCM image analysis in the daily activities of confocalists. Automated image analysis systems may provide great assistance in the decision-making process of RCM examiners looking at skin tumors in the future.

These algorithms need to be further developed and thoroughly validated through large randomized controlled trials. This would lead to improved patient care and safety and an enhancement in dermatologists’ productivity.

## Figures and Tables

**Table 1:** Artifact detection in RCM images using various methods of AI.

Study	Sensitivity	Specificity
Bozkurt et al. [27]	76.16%	97.44%
Kose et al. [26]	82%	93%
Kose et al. [30]	83%	92%

RCM: reflectance confocal microscopy; AI: artificial intelligence.

**Table 2:** Artificial intelligence algorithms for reflectance confocal microscopy image analysis.

	Method	Result	Accuracy/Sensitivity and Specificity
Quality assessment of RCM mosaics	MUNet network MED-Net neural network	Differentiate diagnostically relevant patterns (meshwork, ring, nested, artifact).	73% accuracy [27]
		Estimate the quality of RCM composite images (mosaics).	87% accuracy [31]
Automated identification of the DEJ	Texture segmentation, pattern classification, and sequence segmentation	Tile classification for epidermis and dermis in dark skin	90% accuracy [22]
		Identifying epidermis	90% accuracy [32]
		Identifying dermis	76% accuracy [32]
	SVM classification model	Classifying meshwork, ring, clod, specific, and background patterns	55–81% sensitivity and 81–89% specificity [21]
	Per-pixel segmentation	Identifying DEJ	85.7% accuracy [34]
Skin lesion identification	CNN	Identifying lentigos	−81.4% sensitivity and 88.8% specificity [48] −96.2% sensitivity and 100% specificity [49] −81.4% sensitivity and 83.3% specificity [51]
	CART	Identifying melanoma	93.6% accuracy [1]
		Identifying nevi	90.4% accuracy [1]

RCM: reflectance confocal microscopy; SVM: support vector machine; DEJ: dermal-epidermal junction; CNN: convolutional neural network; CART: classification and regression trees.

## Data Availability

Not applicable.
